# Exosome: A New Player in Translational Nanomedicine

**DOI:** 10.3390/jcm9082380

**Published:** 2020-07-26

**Authors:** Houssam Aheget, María Tristán-Manzano, Loubna Mazini, Marina Cortijo-Gutierrez, Pablo Galindo-Moreno, Concha Herrera, Francisco Martin, Juan Antonio Marchal, Karim Benabdellah

**Affiliations:** 1 Genomic Medicine Department, GENYO, Centre for Genomics and Oncological Research, Pfizer-University of Granada (Andalusian Regional Government), Health Sciences Technology Park, Av. de la Illustration 114, 18016 Granada, Spain; houssam.aheget@genyo.es (H.A.); maria.tristan@genyo.es (M.T.-M.); marina.cortijo@genyo.es (M.C.-G.); francisco.martin@genyo.es (F.M.); 2 Medical Application Interface Center, Mohammed VI Polytechnic University, 43152 Ben-Guerir, Morocco; Loubna.MAZINI@um6p.ma; 3 Oral Surgery and Implant Dentistry Department, School of Dentistry, University of Granada, 18011 Granada, Spain; pgalindo@ugr.es; 4 Maimonides Institute of Biomedical Research in Cordoba (IMIBIC), 14004 Cordoba, Spain; inmaculada.herrera.sspa@juntadeandalucia.es; 5 Department of Haematology, Reina Sofía University Hospital, 14004 Cordoba, Spain; 6 Biomedical Research Institute, ibs. Granada, 18012 Granada, Spain; jmarchal@ugr.es; 7 Biopathology and Regenerative Medicine Institute (IBIMER), Centre for Biomedical Research (CIBM), University of Granada, 18016 Granada, Spain; 8 Department of Human Anatomy and Embryology, Faculty of Medicine, University of Granada, 18016 Granada, Spain; 9 Excellence Research Unit Modeling Nature (MNat), University of Granada, 18016 Granada, Spain

**Keywords:** immunotherapy, exosomes, CARs, gene editing, cancer, liquid biopsies

## Abstract

Summary: Exosomes are extracellular vesicles released by the vast majority of cell types both in vivo and ex vivo, upon the fusion of multivesicular bodies (MVBs) with the cellular plasma membrane. Two main functions have been attributed to exosomes: their capacity to transport proteins, lipids and nucleic acids between cells and organs, as well as their potential to act as natural intercellular communicators in normal biological processes and in pathologies. From a clinical perspective, the majority of applications use exosomes as biomarkers of disease. A new approach uses exosomes as biologically active carriers to provide a platform for the enhanced delivery of cargo in vivo. One of the major limitations in developing exosome-based therapies is the difficulty of producing sufficient amounts of safe and efficient exosomes. The identification of potential proteins involved in exosome biogenesis is expected to directly cause a deliberate increase in exosome production. In this review, we summarize the current state of knowledge regarding exosomes, with particular emphasis on their structural features, biosynthesis pathways, production techniques and potential clinical applications.

## 1. Introduction

Extracellular vesicles (EVs) are differently sized vesicles released by the vast majority of cell types both in vivo and ex vivo. Two main functions have been attributed to EVs: (1) their capacity as natural intercellular communicators to transport proteins, lipids and nucleic acids between cells and organs in normal biological processes and (2) their active involvement in the progression of pathologies such as cancer. Based on their size, biogenesis pathways and other biophysical and biochemical criteria, EVs can be grouped into two main categories: microvesicles (MVs; 100–1000nm) and exosomes (EXOs; 30–100 nm) [1,2,3].

**Figure 1:**
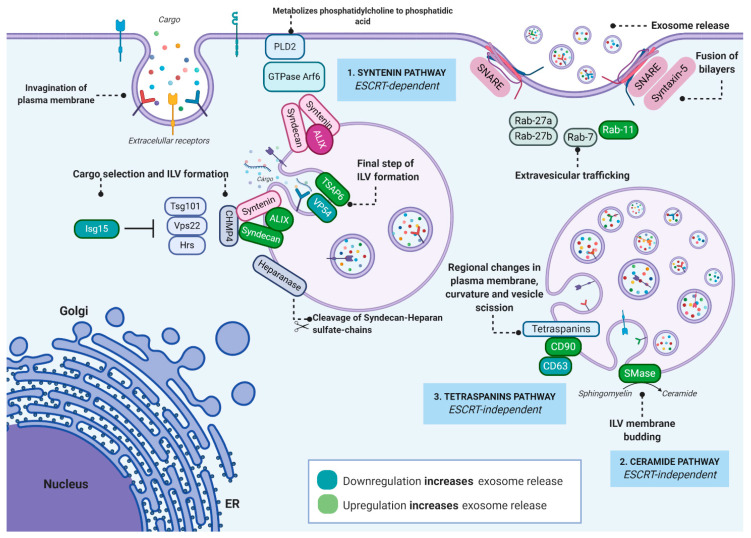
Different exosome biogenesis pathways. Exosome formation begins with syntenin-syndecan interactions which require direct interaction between ALIX and CHMP4 proteins. The intervention of two additional components, Tsg 101 (ESCRT-1) and Vps22 (ESCRT-II), has also been reported, although their mode of action remains little understood. Exosome formation is further regulated by heparanase, an enzyme that cleaves syndecan heparan sulfate, while the small GTase Arf6 also plays a crucial role. The small GTPase ADP ribosylation factor 6 (Arf6) and its effector phospholipase D2 (PLD2) regulate the syntenin pathway. The interaction of Arf6 and PLD2 affects exosome formation by controlling the budding of intraluminal vesicles (ILVs) in multivesicular bodies (MVBs). The silencing of hepatocyte growth-factor-regulated tyrosine kinase substrate (Hrs)proteins, which interact with the tumour susceptibility gene 101 (tsg101) in exosome biogenesis, decreases the number of vesicles [31]. As interferon-stimulated gene 15 (Isg15) expression inhibits Tsg101 ubiquitination, the disruption of tsg15 may increase exosome release. The upregulation of the tumor-suppressor-activated pathway 6 (TSAP6), a p53-inducible transmembrane protein, has been shown to increase exosome production [32]. Two other possibilities are involved in ESCRT-independent pathway: the ceramide-based sphingomyelinase (SMase) pathway, in which sphingomyelin is hydrolysed into phosphorylcoline, and ceramide, which contributes to alternative exosome production. The third pathway is a tetraspanin-dependent pathway that involves CD63, belonging to the superfamily of tetraspanins, which, along with their partner molecules, form tetraspanin-enriched microdomains that contribute to exosome formation. Furthermore, exosome trafficking is regulated by the small GTPase, a member of the Rab and Ral protein superfamilies. For instance, Rab11, together with Rab27a/b, facilitate exovesicular secretion in a calcium-dependent manner [33]. Finally, SNARE and syntaxin 5 proteins enable vesicles to dock and fuse with the plasma membrane and to release exosomes into the external medium.

Microvesicles (MVs) can be distinguished from other EVs by their size and formation mechanisms, including cytoskeleton remodelling and phosphatidylserine externalization [4,5]. Like other EVs, MVs are derived from several cell types (Figure 1). Their formation is stimulated under specific conditions, by inflammatory processes and hypoxia among other stimuli [6,7,8,9,10,11,12], and they generally maintain the original cell-surface-specific antigens [13,14,15,16,17,18,19]. MVs play several physiological roles in the body through the transfer of active molecules, such as microRNA, proteins and lipids. These different functions enable MVs to regulate cellular processes including intercellular immune responses [20,21] angiogenesis [22], neuronal regeneration [23], anti-inflammatory protection [21] and coagulant mediation [24]. In addition to physiological processes, EVs are involved in intracellular degradation systems such as autophagy through specific signalling pathways [25,26,27] and the activation of molecules involved in apoptotic pathways [28,29,30]. Given the characteristics described above, MVs are clearly not just simple by-products of physiological and pathological processes, but are also key players in many different pathways. Here, we review the current state of knowledge concerning exosomes which are not directly shed from the parent cell plasma membrane, but rather are formed through a more complex process, with particular emphasis on their structural features, biosynthesis pathways, production techniques and potential clinical applications.

## 2. Exosome Biogenesis, Regulation and Function

### 2.1. Exosome Biogenesis

Unlike MVs, exosomes constitute some of the most sophisticated intracellular trafficking systems (Figure 1). Exosome biogenesis takes place via plasma membrane (PM) invagination to form endosomes through the fusion of several primary vesicles. The maturation process occurs during the intracellular trafficking of endosomes from the PM to the centre of the cell, leading to overall changes in the lipid and protein composition of their cargo. In this regard, more than twenty proteins are involved and distributed through four Endosomal sorting complexes required for transport ESCRT (ESCRT-0, ESCRT-I, ESCRT-II and ESCRT-III), which complement the ESCRT-independent mechanism. Both pathways, which work synergistically, are involved in (1) protein sequestration and modification, (2) the processing and trafficking of the resulting vesicles, and (3) their fusion to the plasma membrane [33]. These mechanisms have been the subject of intense research in recent years. However, due to experimental challenges, it is unclear at which step the different molecules and enzymes are involved. Below, we discuss the different pathways involved in exosome biogenesis, sorting and release. We will also focus on proteins which may boost the production of exosomes or modulate their surface and content. Some non-ESCRT proteins, sphingolipids (SLs) and their metabolic enzymes will be highlighted as potential targets and as a new strategy to amplify exosome production.

The components of the first protein complex, ESCRT-0, are the hepatocyte growth-factor-regulated tyrosine kinase substrate (Hrs), the signal-transducing adaptor molecule (STAM) and tumor susceptibility gene 101 *(TSG101)*. This complex concentrates ubiquitylated cargo at specific micro-domains, thus facilitating the first step in membrane invagination [34]. This first phase involves a group of proteins, ALG-2-interacting protein X, *(ALIX) syntenin and syndecan*, which are involved in intraluminal vesicle (ILV) formation and cargo selection [35]. Depletion of ALIX proteins increases the amount of MHC-II in cells and, thus, in secreted vesicles, indicating that, rather than regulating vesicle biogenesis, ALIX proteins alter the exosomal membrane. This suggests that these proteins may be good candidates to modulate surface expression [36]. Other well-characterized proteins are syndecans (SDCs), type-I integral membrane heparan sulphate proteoglycans (HSPGs), composed of four genes (SDC 1-4). SDC 4 regulates several vesicular trafficking pathways together with syntenin and the adaptor protein Bro1/ALIX [37]. Transmembrane protein tumour-suppressor-activated pathway 6 (TSAP6), also identified as ferrireductase Steap3, plays a fundamental role during the first steps of the biogenesis pathway. This protein is strongly activated by DNA damage-activated transcription factor p53 in several cell lines [38,39,40]. TSAP6-deficient mice exhibit a phenotype associated with abnormal reticulocyte maturation, a process known to be dependent on exosome secretion [41].

As mentioned above, several studies have highlighted the existence of at least two ESCRT-independent mechanisms in the initial steps of exosome formation involving lipids and tetraspanins. These protein superfamilies, together with a wide variety of transmembrane and cytosolic proteins, mediate the organization of tetraspanin-enriched microdomains (TEMS) in the plasma membrane and the biogenesis of exosomes [42,43]. Tetraspanins CD9, CD63, CD81, CD82 and CD151 are widely distributed among the different cell types, while others, such as Tssc6 CD37 and CD53, are restricted to specific tissues [44]. Exosome release by dendritic cells generated from CD9 knockout mice has been demonstrated to be lower than that from wild-type [45]. The knockdown of tetraspanin protein CD63 induces a significant increase in exosome production, thus confirming the important role played by CD63 in multivesicular endosome (MVE) biogenesis and/or trafficking [46]. The second ESCRT-independent mechanism involves ceramide, a simple sphingolipid (SL), which plays a critical role in membrane biogenesis [47]. SL biosynthesis starts in the endothelial reticulum (ER) with the condensation of L-serine and palmitoyl-CoA which are catalysed by serine palmitoyl-transferase (SPT), leading to the generation of a variety of long-chain SL bases. The resulting product is reduced by 3-ketosphinganine reductase and N-acylation, followed by a final reduction in dihydroceramide to ceramide mediated by dihydroceramide desaturase [48]. Ceramide can also be generated by sphingolipid hydrolysis through the intervention of sphingomyelinases (SMases) [49].

### 2.2. Regulation and Function

As mentioned above, as exosomes are highly sophisticated vesicles involved in numerous pathological and physiological processes, their secretion is strictly regulated and influenced by external and internal stimuli including biotic and abiotic stresses [50]. Trafficking events are governed by the Ras-associated binding (Rab) GTPase. Rab family proteins regulate the traffic pathways of different membrane compartments including exosomes. Rab27-deficient mice have been shown to be defective in several membrane-associated processes such as improper neutrophil chemotaxis. Rab27a-deficient natural killer (NK) cells and cytotoxic T lymphocytes (CTLs) exhibit impaired cytotoxic granule exocytosis [51,52] and granule platelet release [53,54]. Five potential Rab GTPases, Rab2b, Rab5a, Rab9a, Rab27a and Rab27b, have been identified in HeLa cells during shRNA screening, targeting 59 GTPases, which play a major role in exosome secretion [55], with the involvement of Rab27a and Rab27b in exosome biogenesis attracting particular attention. A recent study reports that Rab27b, rather than Rab27a, regulates exosomal secretion in human umbilical vein endothelial cells (HUVECs) [56]. This and other studies have observed that Rab11 depletion severely diminishes exosome secretion in several cell types and have highlighted the role of Rab family proteins in exosome biogenesis.

The Rho/Rac/cdc42 family of small membrane GTPases is also involved in the exosome pathway, with the RhoA effector citron kinase, in particular, observed to have a positive effect on exosome release [57]. The fusion of the resulting membrane with the plasma membrane (PM), as well as the release of exosomes to the extracellular medium, are both regulated by N-ethylmaleimide-sensitive-factor attachment receptor (SNARE) proteins [58]. Two SNARE protein family members, vesicular associated membrane protein 7 (VAMP7) and Synaptobrevin homolog YKT6 (YKT6), have been identified to play a major role in exosome release, specifically in human lung cancer cell lines, human embryonic kidney 293 cells (HEK293) and Adenocarcinomic human alveolar basal epithelial 549 cells (A549) [59,60].

External factors, such as the impact of viral infection on exosome production through mechanisms including the regulation of specific proteins, are closely associated with exosome biogenesis [61,62,63]. The hepatitis A virus (HAV) can hijack ALIX exosome-like pathways [64,65], while the respiratory syncytial virus (RSV) uses the exosome cargo to inhibit immune responses in the course of viral infection [66]. Bacterial and parasitic infections also affect exosome production and secretion [67,68], while metabolic dysfunction due to abiotic stress can also lead to exosomal changes. For example, environmental stresses, including ionizing radiation, can alter exosome secretion, composition and function [39,69,70,71,72].

## 3. Different Types and Functions of Cells that Release Exosomes

Exosomes can be produced by the vast majority of cells with different origins and numerous functions. The cells from which exosomes are secreted include T cells [73,74], platelets [75], megakaryocytes [76] mast cells [77,78], neurons [79,80], oligodendrocytes [81] and Schwann cells [82,83,84,85]. Similarly, cells with stemness properties, such as mesenchymal stromal cells (MSCs) [86,87,88] and induced pluripotent stem cells (iPSCs) [89,90], have been reported to release exosomes. In addition, exosomes are found in biological fluids including plasma [91,92,93,94], urine [95,96,97] saliva [98], amniotic fluid [99] and breast milk [100]. In the sections below, we provide details of major cells that play a key role in exosome secretion in different metabolic and pathological pathways.

### 3.1. Exosomes Derived from Mesenchymal Stem/Stromal Cells of Different Sources Involved in a Wide Range of Diseases and Metabolic Pathways

Mesenchymal stromal cells (MSCs) are resident adult stem cells that have been identified in virtually all human tissues including bone marrow (BM), peripheral and cord blood (CB), dental pulp, liver and skin [101]. This explains their critical role in tissue repair and regeneration despite the differences observed in their population numbers, cell profiles and proliferation rates [102,103]. MSCs are characterized by self-renewal and differentiation capacity both in vitro and in vivo. However, their plasticity and heightened capacity to differentiate into endoderm and ectoderm cell layers have made MSCs of great interest for cell-based therapies in the field of regenerative medicine [104,105,106]. Although MSCs are known to be mediated through cell-cell communication, their secretome-rich cytokines, chemokines, micro-RNA, as well as different growth factors, involved in biological pathways, including cell proliferation, differentiation, migration and senescence [107,108], make them particularly suited to use in cell-based therapies. These secretomes are made up of extracellular vesicles (EVs) including exosomes [109,110,111], which can be characterized using specific International Society for Cellular Therapy (ISCT) guidelines. According to the ISCT, while positively expressing the stromal markers CD73 and CD105, MSCs negatively express the hematopoietic markers CD14, CD34 and CD45 [112]. MSCs are plastic-adherent cells of a fibroblast-like morphology capable of long-term expansion in culture and tri-lineage differentiation potential into osteogenic, adipogenic and chondrogenic progenitors. However, their immunogenicity is the most important reason for using MSCs in cell-based therapies. MSCs are immunosuppressive and inhibit T cell activation due to the lack of major histocompatibility complex (MHC) II [113,114]. Given the impaired expression of CD80 and CD86 in dendritic cells, in addition to B cell proliferation and differentiation, MSCs offer great potential for use in allogeneic transplantation [115,116]. Despite the usefulness of these criteria for identifying MSCs, these cell populations are reported to be heterogenous with regard to their non-clonal proliferation, differentiation potencies, stromal stem cell profiles and committed progenitors [117,118]. These differences appear to be related to their tissue origin and to the cell separation and culture expansion techniques used [119,120]. Thus, the complex composition of the exosomes released is markedly influenced by initial local cell crosstalk and microenvironmental priming.

Despite clinical successes, consistency and safety issues remain a matter of debate [86,121]. MSC-derived exosomes present in conditioned media are considered an alternative to MSC-based therapies due to their superior efficiency and scalability [122]. More than 200 preclinical studies have been published as of July, 2020, on the applications of MSC-derived exosomes to a wide range of pathologies including neurological, cardiovascular, immunological and kidney diseases (https://clinicaltrials.gov/). MSC-derived exosomes obtained from different sources, particularly human bone-marrow (hBM) and human umbilical cord perivascular cells (hUCPVCs), have proven to have an impressive effect on neurological tissues, blood–brain barrier stability in lipopolysaccharide-induced neuroinflammation and on reactive astrogliosis [123,124,125]. Emerging evidence suggests that neurological disorders can be successfully treated by exosome-based therapy when the auto-regenerative capacity of the central nervous system (CNS) is limited [126,127,128]. BM-MSC-derived exosomes and human umbilical cord (hUC) MSC exosomes are also effectively used for cardiac tissue neovascularization following ischemic injury [129] and for endothelial function enhancement, respectively [130,131]. Similarly, liver function is ameliorated by MSC-derived exosomes through the epithelial–mesenchymal transition (EMT) of hepatocyte and collagen production and through serum aspartate aminotransferase restoration [132,133,134,135].

### 3.2. Exosomes Derived from Immune System-Related Cells That Play a Key Role in Several Immunological Processes

**Figure 2:**
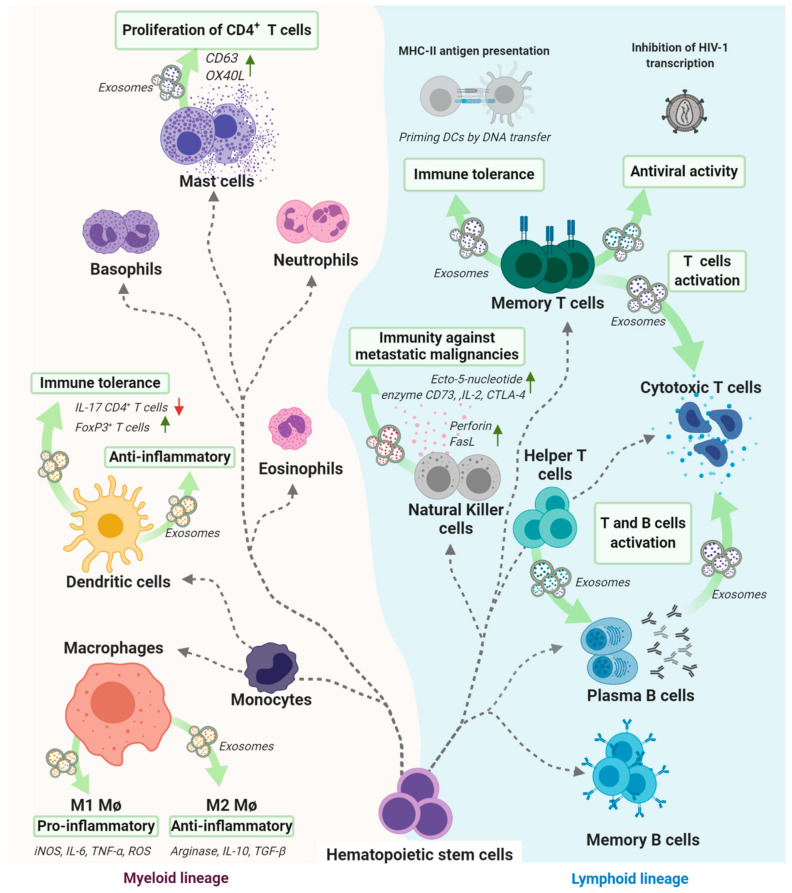
Secretion of exosomes associated with immune cells types and their modes of action.

Many immune cells, including T and B cells, macrophages, natural killers (NKs) and dendritic cells (DCs), are associated with exosome secretion capacity [136]. The existence of a set of proteins, such as CD63, Major histocompatibility complex class II (MHC-II), Fas ligand (FasL) and T cell receptor (TCR) (http://www.exocarta.org), on cellular surfaces provide further evidence of their role as mediators, modulators and activators in the immune system [137,138]. T cells, including CD4^+^ helper and CD8^+^ cytotoxic T cells, as well as regulatory T cells (Tregs), secrete exosomes and play different roles depending on T cell subtype origin and activation status (Figure 2). For example, exosomes derived from stimulated T cells can act as autologous signals to increase the proliferation of resting cells, resulting in an altered cytokine secretion profile [139]. Unlike classical T cells, exosomes derived from chimeric antigen receptor (CAR) T cells have recently been found to provide relatively safer therapies [140,141]. On the other hand, exosomes derived from the more antigen-specific subtype CD8^+^ T cell show antiviral activity associated with membrane proteins secreted via exosomes [142]. Moreover, CD4^+^-associated exosomes have been reported to significantly boost B cell activation, proliferation and, thus, antibody production [143,144] and to act as immunoregulators [145]. Exosomes secreted by CD4^+^CD25^+^Foxp3 Treg cells, a subset of CD4^+^ T cells, specializing in immune tolerance establishment and maintenance, which use a diverse set of mechanisms to enforce peripheral tolerance, are thought to be deeply involved in immune regulation [146]. In the murine model, Tregs appear to produce quantitatively more exosomes than naïve CD4^+^ and CD8^+^ T cells [147]. Treg-derived exosomes also express the cell-surface enzyme CD73[148,149], which suppresses immune responses [150], the IL-2 receptor chain CD25, which plays a key role in autoimmune disease suppression [151], and T Lymphocyte-associated antigen-4 (CTLA-4), an immune inhibitory factor constitutively expressed in Tregs [152].

Both lymphoid and myeloid lineages have the capacity to secrete exosomes. Several studies have highlighted the potential of B cells to secrete exosomes carrying the peptide pMHC-II, in addition to costimulatory and adhesion molecules. This type of exosome induces antigen-specific MHC class II-restricted T cell responses [153]. The second potential target of B-cell-derived exosomes is dendritic cells (DCs) which present MHC-II peptides to CD4^+^ T cells, T cell-derived exosome-DC reciprocal interactions begin with DC priming by exosomes which contain genomic and mitochondrial DNA through antigen-driven contacts [154]. Exosomes derived from a specific subset of T cells may regulate other T cell subtypes. In addition, T-cell-derived exosomes specifically inhibit viral transcription through the presence of antiviral membrane-bound factors [142]. Mast cells constitutively release exosomes expressing CD63 and OX40 ligand (OX40L), which promote CD4^+^ T cell proliferation, thus facilitating T helper 2 (Th2) cell differentiation [155]. DCs are also able to secrete exosomes which express, on their surface, MHC-peptide complexes, T cells costimulatory molecules and other compounds which interact with immune cells. Furthermore, macrophages secrete exosomes with proinflammatory activity when secreted by M1 macrophages (M1) and with anti-inflammatory activity when secreted by M2 macrophages (M2).

Natural killer (NK) cells, members of the lymphoid cell family, play a major role in innate immunity and tumor progression control through their cytolytic activity, cytokine production and by improving T-helper 1 responses. Exosomes produced from NK cells and their apoptotic activity in tumor cells have also been studied. Exosomes obtained from IL-2- and IL-15-stimulated NK cells were detected in peripheral blood expressing the typical NK-related molecules CD16, CD69 and NKG2D which have high penetrance rates and a marked cytolytic effect on tumor sites [156]. NK-derived exosomes carrying the tumor suppressor microRNA (miR)-186 exhibit potent activity against neuroblastoma cell lines [157] and aggressive melanoma in vitro and in vivo [158], thus opening up the possibility of clinical applications using the antitumor activity of NK-derived exosomes [159]. Dendritic cells (DCs), another powerful cancer immunotherapy tool, have recently become an alternative source of exosomes [160,161]. In fact, several groups have demonstrated the feasibility of obtaining exosomes from DCs [162] to alleviate the clinical symptoms of various diseases [163,164,165,166,167,168]. These exosomes alleviate the effects of hepatic ischemia/reperfusion (I/R) injury by modulating Treg/Th17 cell balance [169] and also induce transplantation immune tolerance [170,171,172].

Finally, macrophages, a diverse cell population found in specific organs and the blood stream, can be divided into two sub-groups: classically activated macrophages (M1) and alternatively activated macrophages (M2), induced by type 1 T helper cells (Th1) and by type 2 T helper cells (Th2), respectively. Despite their proinflammatory profile, M1 macrophages polarize to M2 status given their anti-inflammatory capacity to respond to microenvironmental stimuli during inflammation-associated diseases. M1 macrophages secrete elevated levels of proinflammatory cytokines such as tumor necrosis factor-alpha (TNF-), interleukin (IL)-1 and IL-6; M2 macrophages, composed of four subgroups M2a, M2b, M2c and M2d, secrete immunoregulatory cytokines such as IL-10 [173]. Exosomes recently obtained from both types of these multi-functional macrophages [174], isolated from M2a and M2b, presented strong anti-inflammatory activity, through the Th2 activation and immunoregulation [175]. In addition, M2a-exosomes are capable of regulating the behaviour of breast cancer cells by inducing or reversing their dormancy [176].

## 4. Exosome Manufacturing Status and Challenges

Based on their role in intercellular communication, especially with stem cells in their microenvironment, exosomes are expected to play a critical role in the regulation of numerous physiological and pathological processes. Their use in new disease therapy strategies presents many challenges, mainly with regard to regulatory production guidelines, qualified staff and the marketing strategies required for this widely used type of therapy. Another challenge is the difficulty of classifying exosome-based therapies which differ enormously from cell-based treatments. In terms of size, exosomes are likely to be in the form of platelet lysates, ranging from whole cells to pharmaceutical molecules. Quality control is likely to combine current Good Manufacturing Practice (cGMP) guidelines for cells and traditional cGMP for pharmaceutical drugs [177,178]. European regulatory agencies and the Food and Drug Administration (FDA), classify human use extracellular vesicles (EVs) as biological medications, have laid down a regulatory framework for manufacture and clinical trials in the journal of the International Society for Extracellular Vesicles (ISEV) published recently [179]. In their 2018 guidelines, ISEV states that the following information is required for each EV formulation: (i) precise data concerning origin, including the number of secreting cells, biofluid volume and tissue mass, (ii) precise EVs abundance data, including total particle numbers and/or protein and lipid content, (iii) presence of components associated with EV subtypes and EVs generically, depending on the specific intended function and iv) the presence of non-vesicular and co-isolated components [180].

The heterogenous composition of exosomes, containing mostly proteins and nucleic acids, is subject to potency and quality testing, similar to that used for current cellular therapies [178]. It is of critical importance to define the pharmaceutical classification of active substances responsible for the effects of the therapy in order to determine the pharmaceutical quality control strategy to be used for exosome production [181,182]. These active substances can be overexpressed by genome editing to improve homogeneity, purity and manufacturing reproducibility. The homogeneity assays should also be optimized to track the biomarkers selected in each batch and to identify non-active ingredients.

The general production conditions for these products, which are similar to those for drug manufacturing, include current GMP guidelines, large-scale production characterized by high reproducibility, scalability, stability, storage, banking and clinical quality control in allogenic settings, as reported elsewhere [177,183]. The collection, separation, expansion storage and point-of-care transfer of stem cell progenitors and mature cells need to be integrated into therapeutic strategies and strictly controlled and regulated to ensure patient safety and to maintain the sustainable therapeutic efficacy of the purified exosomes.

### 4.1. Large Scale Production of Exosomes

One of the major challenges in developing exosome-based therapies is the need to produce a sufficient number of safe and efficient exosomes. Depending on the disease and condition of the patient, considerable quantities of exosomes will be required to provide adequate treatment. For example, EVs were administered to a patient with graft-versus-host disease (GVHD) in progressively increasing doses, beginning with a total protein dose of 0.05–0.15 mg/kg and ending with a dose of 0.20–0.60 mg/kg [184]. The appropriate dose of exosome proteins is dependent upon the disease of the patient and associated factors. Exosomes are also extracted from the culture media of large-scale biotechnological waste products [185,186,187]. To boost the production of cell culture exosomes, the expanded stem, progenitor and mature cells need to be adequately activated by growth factors, nutrients, oxygen concentrations and other stimuli. The long-term expansion of MSCs affects their morphology, stem cell-associated profile, proliferation and clonogenic capacity [188], as well as the links between cell profile and physiological changes. Some evidence indicates that early expansion of MSCs is related to their stemness profile, while long-term expanded MSCs are stromal cells associated with a senescent phenotype [188,189]. Recent studies report increasing proliferation induced by younger MSC-derived secretome, probably due to the secretion of rejuvenating growth factors such as GDF11 [190,191]. Interestingly, other studies have suggested that the secretome of senescent MSCs has a biological effect on their microenvironment [103,189,192,193]. This senescence messaging secretome, or senescence-associated secretory phenotype (SASP), probably induces changes in the cellular transcriptional program [194] which, in turn, lead to changes in the number and composition of EVs, thus reflecting the senescent profile of the parent MSCs [192,193,195]. SASPs have also been reported to play an effective role in inducing senescence in immortalized prostate cells [193]. Alessio et al. (2019) have also identified an increase in IGFBP-4 protein levels in the SASP caused by genotoxic stress and initial senescence status [196]. Thus, to better adapt exosomes to their intended therapeutic function, SASPs should be avoided during culture production. Nevertheless, MSC culture expansion is critical for pooling the appropriate number of cells, mainly in closed-culture bioreactors, suggesting that the avoidance of SASPs during exosome production has become an important issue. The use of mesenchymal hematopoietic and endothelial markers to sort cells could be useful for purifying specific MSC populations. However, as with bone marrow (BM)-MSCs, initial MSC identification of the sorted subpopulations could limit yields and availability. Gene editing could therefore be essential to increase the number of exosomes produced per MSC with reduced cell culture throughput, thus avoiding SASP development during long-term culture expansion.

Another technical hurdle is to be surmounted is the need to reduce artifacts when using differential ultracentrifugation [197], gel-filtration on special matrices [198] and size-exclusion chromatography (SEC) for exosome separation and concentration. These techniques have recently been reported to completely transform the basic composition of exosomes [199]. However, tangential flow filtration (TFF) and SEC purification, which appear to be best adapted to large-scale production [200], are used commercially to produce recombinant proteins and antibodies, methods which might also be suitable for exosome purification. For enhanced specificity, additional techniques are combined with primary steps such as washing and ultrafiltration [201,202,203]. High-resolution density gradient fractionation and direct immunoaffinity capture can also be used for EV analysis and characterization [204].

### 4.2. High-Quality Uniform Exosomes

MSC-derived exosome therapies are largely dependent upon the regenerative and immunomodulatory capacities of MSCs. Some evidence shows that hypoxia, nutrient starvation and microenvironment changes in pH enhance the release of EVs such as exosomes [204]. Exosomes can also be engineered to take advantage of their natural production processes and properties combined with genetic and non-genetic techniques to add new functionalities. Different active procedures are used to selectively enrich exosome cargo with miRNAs or small-molecule drugs. A poly (A)-binding protein is used to selectively recruit mRNAs into exosomes, while a zipcode-like 25 nucleotide (nt) sequence can be incorporated into the three prime untranslated region (3′UTR) of the mRNA of interest and be recruited by Z-DNA binding protein 1 (ZBP1) [205]. Receptor–ligand pairs are also used to deliver modified exosomes presenting membrane-bound ligand receptors to target surface cells [206,207]. Exosomes can also be enhanced by active loading through electroporation [208] and chemical conjugation [209]. These molecular biology techniques raise the expression of the protein and nucleic acid of interest well above physiological expression levels. However, the production of uniform exosomes with regard to their composition, membrane markers and even size, is difficult but not impossible. Further research is critical to improve molecular targeting in different aspects of exosome biogenesis and its involvement in biological processes. Interestingly, exosomes can also be engineered to lack class II MHC transactivators or major histocompatibility complex (MHC) genes to prevent the risk of immunogenicity facilitating allogeneic exosome use [210].

MSCs can also be primed with inflammatory cytokines such as Interferon gamma (IFN-γ) and TNF-α to produce EVs that are more immunosuppressive than control cells [211]. These vesicles enable the proliferation levels of NK cells, as well as of T and B lymphocytes, to be reduced. When cultured under hypoxic conditions, MSC-derived EVs become potent anti-inflammatory agents by inducing polarization from M1 to M2 macrophages [212]. Nevertheless, genetic modification appears to be more precise for overexpressing specific therapeutic factors and for cell priming. Other concerns include the need to perform stem cell expansion with serum-free media given constitutively distinct serum batches which influence cell proliferation and function. In addition, serum contains EVs that might affect cell responses and activity in the recipient [213].

### 4.3. Storage Conditions

During bioprocessing, the production of homogenous exosome batches for effective, stable, reproducible and successful chemically defined drugs widely used in new therapies, presents a major challenge. The scalability of the final product is thus contingent upon storage conditions and the packaging used prior to administration to the patient. However, cold chain and supply chain management needs to be consistent and reliable, while compliance with standards, especially in countries receiving cellular products and derivatives, also needs to be enforced in both manufacturing facilities and clinical sites. Point-of-care infrastructure can impact exosome therapies and their outcomes. In addition, both preclinical and clinical studies of the frequency, stability and administrative clearance procedures for these therapies are required. This will play a critical role in designing formulation strategies and pave the way for widespread use of exosomes for treating diseases. Cryopreservation solutions are mainly designed to meet critical quality requirements, such as viability and potency, for cellular products. The use of cryoprotectants for short- and long-term storage of processed exosomes at very low temperatures (liquid nitrogen) is of primordial importance. Functional tests must be developed and standardized to identify adequate cryoprotectants and appropriate storage times and methods and to evaluate their effects on exosome treatment activity and potency. Fresh or frozen/thawed exosomes can be lyophilized for protective purposes and later reconstituted for transfer from the manufacturing facility to the clinical setting. Such a delivery system would overcome concerns regarding cold-chain preservation.

### 4.4. Quality Control

The stricter 2018 guidelines laid down by ISEV include those regarding EV quantification. Prior to purification, exosome sources must be quantitatively estimated with respect to number of cells, fluid starting volumes and weight/volume/size of tissue used. As no single method is used, total protein amount, total lipid and total particle numbers are commonly measured to quantify exosomal cargo. According to ISEV, protein:particle, lipid:particle and lipid:protein ratios must also be determined for a more precise and reliable quantification of exosomes and EVs, as well as their global purity [180].

Different characterization and validation methods have been developed to analyse single EVs for research and clinical purposes. High-resolution visualization, providing information on vesicular structure and composition, as well as single-particle analysis, with its high-powered biophysical and statistical capabilities, have also been developed [180]. Other methods include transmission electron microscopy (TEM), scanning electron microscopy (SEM), atomic force microscopy (AFM), nanoparticle tracking analysis (NTA), dynamic light scattering (DLS), resistive pulse sensing (RPS), enzyme-linked immunosorbent assays (ELISAs), flow cytometry (FCM), fluorescence-activated cell sorting (FACS), microfluidics and electrochemical biosensors (ECBs) [180]. EV components can be used as specific markers to identify EV subtypes and for further quantification. To determine the presence of lipid bilayers associated with EVs and the exosome membrane, at least one transmembrane or GPI-anchored extracellular protein needs to be identified in the exosome preparation such as non-tissue-specific tetraspanins (CD63, CD81, CD82), multi-pass membrane proteins (CD47, heterotrimeric G proteins GNA*), class I MHC proteins (HLA-A, A/B/C, H2-K/D/Q), integrins (ITGA/ITGB) and transferrin receptors (TFR2). Other specific cell/tissue proteins must be checked: CD3 for T cells, CD37and CD53 for leukocytes, CD9 (negative for NKs, B cells and some MSCs), platelet and endothelial cell adhesion molecule 1 (PECAM1) for endothelial cells, CD45 for immune cells, CD41 and CD42a (GP9) for platelets, glycophorin A (GYPA) for red blood, as well as CD14 for monocytes and class II MHC proteins (HLA-DR/DP/DQ, H2-A). At least one cytosolic/periplasmic protein associated with lipids, such as heat shock protein Hsp70 (HSPA1A), actins (ACTs), tubulins (TUBs), glyceraldehyde 3-phosphate dehydrogenase (GAPDH) enzymes and those associated with membrane protein-binding capacity must also be characterized [180], which are commonly quantified using the Western blotting method. Exosomes are also rich in sphingomyelin, phosphatidylserine (PS), cholesterol and saturated fatty acids [214]. The ganglioside GM3, ceramides and their derivatives are also rich in exosomes [215], while sphingomyelin, cholesterol and GM3 have the capacity to enhance the rigidity and stability of the exosomal membrane [216]. These lipids can also act as biomarkers for exosome characterization.

RNA can be quantified using profiling and capillary electrophoresis tools in global RNA assays [217] or dyes [218]. As RNA binds to co-separated circulating proteins or other particles, specific measurements are difficult to perform and do not provide purity data on all EV subtypes. Exosome purity is a particular concern, as most characterizations of EVs only evaluate positive markers, and, in only a few studies are positive markers combined with negative markers to analyse and track co-isolated non-EV components. Lipoproteins are the principal separated components present in serum, plasma and urine, protein, protein/nucleic acid aggregates and ribosomal proteins in non-EV co-isolated components. The measurement of other components in intracellular compartments, such as mitochondria, nuclei and those associated with secretory pathways, provides crucial information on EVs and exosome purity for future therapies.

### 4.5. Treatments for Humans

Although most clinical therapies use exosomes as biomarkers of disease, several preclinical and clinical studies use exosomes therapeutically, either as biologically active carriers to enhance in vivo cargo delivery, or as immune system activators, among other approaches. Given that exosomes can mediate intercellular communication by delivering messages from parent cells to acceptor cells in the form of mRNA, siRNA, miRNA and proteins, exogenous siRNA can be directly loaded into exosomes using electroporation, resulting in strong mRNA and protein knockdown of targeted genes in animal brain [219,220,221] and hepatocarcinoma cells (HCCs) [222]. The antitumoral therapeutic effects of exosomes are partly due to the release of exosomes by activated antigen-presenting cells, such as DCs, macrophages, T lymphocytes and B cells, which package cellular components from cancer cells and subsequently induce antitumoral responses by presenting tumor antigens to immune cells [223]. For instance, DC-secreted exosomes incubated with human breast adenocarcinoma cells (SK-BR-3) trigger tumor-sensitized T cells in order to secrete high levels of interferon-γ (IFN-γ) to enhance cancer immunotherapy [224]. T-cell-derived EVs modulate endothelial cell responses to the vascular endothelial growth factor (VEGF) and alter tube formation and gene expression in target endothelial cells. Furthermore, T-cell-released exosomes have been shown to destroy tumor stroma and to prevent tumor invasion and metastases [225]. Exosomes are also considered strategic partners and even substitutes in chimeric antigen receptor T cell (CAR-T) therapy, especially with regard to solid tumor approaches. Given their properties, CAR-T cell-derived exosomes have great potential as cancer killers in CAR-T cell-free mediated therapies. In addition, CAR-T cell-induced toxicity can be controlled by using CAR-T exosomes as powerful serial killers of tumor cells to replace immune cells [140]. In addition to the pre-clinical trials carried out up to June 2020, 14 clinical trials focusing on exosomes as therapeutic agents are actively recruiting (Table 1). Recently, a pilot study of MSC-derived exosomes for treating severe novel coronavirus pneumonia (NCP) patients was carried out, following previous experimental studies showing that MSCs and MSC-Exo significantly reduce lung inflammation and other clinical manifestations (NCT04276987; NCT04389385). Although central nervous system (CNS) diseases have also been successfully treated using MSC-derived exosomes, the delivery of other drugs targeting inflammatory tissues, to the brain still presents major challenges due to limited penetrance. Given this drawback on the one hand and the capacity of exosomes to be taken up by immature myeloid cells on the other, several exosome therapies targeting brain tissues have been moved forward to clinical trials. Thus, exosomes loaded with anti-inflammatory agents were delivered by intravenous, intracerebral and intrathecal administration, which penetrated the blood–brain-barrier (BBB) effectively to treat neuralgia and refractory depression (NCT04202783, NCT04202770). Clinical trials evaluated the effects of autologous plasma-derived exosomes (NCT02565264) following previous reports that serum exosome levels decreased significantly in chronic multi-system autoimmune disorders such as systemic sclerosis. Other recent clinical trials focused on the critical effects of MSCs on ischemia-reperfusion injuries to the heart, lungs and others organs; in an ongoing study with promising results (NCT04356300), several patients diagnosed with acute type A aortic dissection (ATAAD) were intravenously administered with exosomes. The immunomodulatory properties of mesenchymal stromal cell (MSC) exosomes were explored in the treatment of dry eye in 27 patients with chronic graft-versus-host disease (cGVHD) in a clinical trial, which suggests that localized administration of MSC-Exo may be effective in treating cGVHD (NCT04213248). The positive effects of a promising multi-pathway exosomal treatment for type 1 diabetes (T1DM) include immune cell response modulation [226], reduced podocyte impairment [227] and induction of proangiogenic properties [228]; given these positive effects, several T1DM patients were treated with MSC-Exo in a clinical test at the El Sahel Teaching Hospital in Cairo, Egypt (NCT02138331). Cancer is already being treated with exosomes which have several advantages over other therapies. For example, given the drug loading capacity of exosomes, a phase I study of MSC-Exo loaded with KrasG12D siRNA to treat metastatic pancreas cancer with KrasG12D mutation was recently launched by the University of Texas M.D. Anderson Cancer Centre in Houston, Texas (NCT03608631); patients will receive MSC-Exo intravenously and outcomes will be evaluated over time.

## 5. Conclusions

Exosome-based therapy is clearly a new player in regenerative medicine and advanced treatments. Initially considered disposable waste, exosomes are now regarded as invaluable genetic information tools, diagnosis markers and therapies. A better understanding of the molecular and cellular processes regulating exosome biogenesis is expected to increase technological advances and potential clinical applications. An immense effort has been made in the last two decades to redefine exosome biogenesis. This basic research highlights the involvement of several groups of proteins and lipids in exosome release and cell surface features which are strictly regulated by various stimuli. It is important to determine whether the resulting exosomes are involved in a complex biological rheostat that fine-tunes downstream biological activity, whether these rheostats are modulated by external stimuli and whether genetic modification enhances exosome release or modifies cargo and surface markers to improve their properties or to prevent rejection. A sophisticated system called EXOsomal Transfer Into Cells (EXOtic) combining six-transmembrane epithelial antigen of prostate 3 (STEAP3) and a syndecan-4 fragment of L-aspartate oxidase, with other genes involved in exosome biogenesis was recently described [229]. The simultaneous expression of these genes significantly boosts exosome production in several cell lines [229]. The multifunctional protein Vacuolar protein sorting-associated protein 4B (VPS4B) belongs to the AAA protein family, involved in lysosomal degradation pathways and intracellular protein trafficking. Thus, the inhibition of VPS4B reduces cellular apoptosis, sheds and repairs the injured cell membrane [230,231] and also increases the secretion of CD63, MHC-II and HSP70 markers associated with exosome secretion [36]. Artificial knockdown of CD63 or VPS4B using existing gene editing methods may be a good strategy to boost exosome production. On the other hand, using existing authorized drugs to interfere with ceramide-dependent exosome biogenesis pathways targeting FMS-like tyrosine kinase 3 (FLT3) [232] or B-cell lymphoma 2 (Bcl-2) [233] may increase ceramide levels and boost exosome release. These drugs should therefore be good candidates for artificial modulation of exosome production. Finally, exosome release is well known to sharply increase, in a Ca^2+^-dependent manner, in hematopoietic cell lines (K562) and in primary neurons and astrocytes [234,235,236]. According to Messenger et al., the exosome release increased five-fold in response to Ca^2+^, an increase mediated by the Munc13-4 Ca^2+^ receptor and Rab-binding proteins [237]. Another major issue is the need to standardize the protocols for biogenesis engineering, stem cell cultures, as well as for exosome purification and storage. However, the uniformity and reproducibility of exosome properties, together with secure production conditions, are expected to be major issues in the coming years. Exosomes, which appear to reflect the biological status and properties of their parent cells, contain cellular biomolecules that are transported to neighbouring organ cells. Although genetic modification can increase exosome secretion, attendant safety hazards cannot be ruled out. Finally, cell priming facilitates the mode of action of exosomes in cell-based therapies.

## Figures and Tables

**Table 1:** A ClinicalTrials.gov search found 12 ongoing national coordinated trials (NCTs) involving exosomes as MSC-based therapeutic agents from different sources and a further 2 NCTs involving plasma-derived and T cell exosomes.

Sponsor, City and State	NCT No	Disease	Exo Source
Wuhan Jinyintan Hospital, Wuhan, China	NCT04276987	Severe novel coronavirus pneumonia	Mesenchymal stem cells (MSCs)
Beni-Suef University, Bani Sweif, Egypt	NCT04270006	Periodontitis	MSCs
Fujian Medical University, Fujian, China	NCT04356300	Multiple organ dysfunction syndrome	MSCs
TC Erciyes University, Talas, Turkey	NCT04389385	Severe novel coronavirus pneumonia	T Cell
Sun Yat-sen University, Guangzhou, China	NCT04213248	Dry eye in patients with chronic graft-versus-host disease (cGVHD)	MSCs
M.D. Anderson Cancer Center, Houston, TX, USA	NCT03608631	Metastatic pancreas cancer with KrasG12D mutation	MSCs
El Sahel Teaching Hospital, Cairo, Egypt	NCT02138331	Type 1 diabetes (T1DM)	MSCs
Ruijin Hospital, Shanghai, China	NCT04313647	Clinical tolerance in healthy volunteers	MSCs
Tianjin Medical University, Tianjin, China	NCT03437759	MSC-Exo promotes MH healing	MSCs
Ruijin Hospital, Shanghai, China	NCT04388982	Alzheimer’s disease	MSCs
Aegle Therapeutics, Arlington, MA, USA	NCT04173650	Dystrophic epidermolysis bullosa	MSCs
Stem Cell and Cancer Institute, Kalbe Farma, Jakarta, Indonesia	NCT04134676	Chronic ulcer wounds	MSCs
Neurological Associates of West Los Angeles, CA, USA	NCT04202783	Craniofacial neuralgia	MSCs
Kumamoto University, Kumamoto, Japan	NCT02565264	Cutaneous wound healing	Plasma
Saeed Oraei Yazdani, Tehran, Iran	NCT03384433	Acute ischemic stroke	MSCs
Neurological Associates of West Los Angeles, CA, USA	NCT04202770	Depression, anxiety and dementias	MSCs
